# Biphasic Regulation of Apoptosis Following Gastric Irreversible Electroporation Using Tissue Immunohistochemistry of Activated Caspase-3 with TUNEL Method

**DOI:** 10.3390/cancers16071389

**Published:** 2024-03-31

**Authors:** Han Jo Jeon, Hoon Jai Chun, Hyuk Soon Choi, Bora Keum, Hong Bae Kim, Jong Hyuk Kim

**Affiliations:** 1 Division of Gastroenterology and Hepatology, Department of Internal Medicine, Korea University College of Medicine, Seoul 02841, Republic of Korea; roadstar82@naver.com (H.J.J.); mdkorea@gmail.com (H.S.C.); borakeum@hanmail.net (B.K.); 2 Department of Biosystems & Biomaterials Science and Engineering, Seoul National University, Seoul 08826, Republic of Korea; ser21@hanmail.net; 3 Department of Small Animal Clinical Sciences, College of Veterinary Medicine, University of Florida, Gainesville, FL 32608, USA; jkim19@ufl.edu

**Keywords:** irreversible electroporation, apoptosis, necrosis, regeneration, ablation, stomach

## Abstract

**Simple Summary:**

Gastric cancer remains the leading cause of death worldwide. Irreversible electroporation is a relatively new ablation technique used for cancer treatment. The present study employed tissue immunohistochemistry to semi-quantitatively assess apoptosis levels in the stomach following irreversible electroporation. This study was designed to understand how changes in the intensity of electrical energy levels influence the expansion and restoration of the apoptotic area within the stomach. As the electrical amplitude increases, the immunohistochemistry of activated cleaved caspase-3 increases, followed by a subsequent decrease, whereas TUNEL staining continues to increase. Masson’s trichrome staining revealed the number of fibroblasts to increase for 7 days, returning to baseline levels by day 14. Apoptosis is biphasically regulated by electrical energy. The efficacy of tumor destruction by irreversible electroporation does not improve linearly with higher electrical intensity; rather, optimal apoptosis arising from the immunological cascade occurs at specific energies. Other electrical parameters should be further investigated.

**Abstract:**

The regulation of apoptosis is the primary goal of ablation therapy. Irreversible electroporation (IRE) is a promising non-thermal tissue ablation-based therapy that induces apoptosis by manipulating electrical conditions. This study aimed to investigate IRE-induced gastric tissue apoptosis in response to changes in the electric field intensity, followed by the repair process. Among the 52 rats used in this study, 24 were used to explore apoptosis, and 28 were used to study regeneration. The apoptosis-to-necrosis ratio of the electrical field strength was evaluated using terminal deoxynucleotidyl transferase-mediated deoxyuridine triphosphate nick-end labeling and caspase-3 immunohistochemistry. The size of IRE-induced ulcers in the gastric tissue continuously increased with increasing electrical intensity (r^2^ = 0.830, *p* < 0.001). The level of apoptosis gradually decreased after peaking at 200 V (1000 V/cm). The size of the 400 V-ablated ulcers continued to decrease, and they were not visible by day 14. The proliferation and migration of epithelial cells with fibroblasts were observed on day 3 and augmented on day 7 post-ablation. This investigation demonstrated the biphasic activation of apoptosis with respect to the electrical field strength. Visually and histologically, IRE-induced gastric ulcers demonstrated complete tissue regeneration after two weeks.

## 1. Introduction

Electroporation is a technique that increases cell permeabilization through the application of an electrical field [1]. The transmembrane electrical pulses produce nano-sized pores in the cell membrane and enhance its permeability. Electroporation can be physiologically classified as either being reversible or irreversible based on the reversibility of temporary cell membrane pores induced by electrical pulses [2]. Reversible electroporation creates transient pores in cell membranes. These pores can recover, allowing cells to maintain their viability and homeostasis, facilitating electrochemotherapy, gene therapy, and drug delivery, and have been shown to be safe and effective [3]. Irreversible electroporation promotes immunogenic cell death by inducing permanent membrane instability through the enlargement of membrane pores [4]. The transition from reversible to irreversible damage, which corresponds to the “point of no return”, is a key focus of therapeutic strategy.

IRE facilitates minimally invasive tissue ablation that selectively eliminates aberrant cells and tissues using multiple, superimposed short-intensity high-voltage electrical pulses [5]. IRE is gaining traction as a novel and emerging biotechnological technique for tissue destruction. The fundamental principle of cancer treatment is to terminate or restrain the uncontrolled growth of the tumor tissue. The primary mechanism of cell death following IRE treatment is membrane injury, initiating apoptosis [6]. Computational simulations and in vivo experiments conducted on swine livers have shown the destruction of tissue when an electrical field strength exceeding 500–600 V/cm is applied [7,8,9]. However, IRE employment for tissues depends on the successful manipulation of electrical parameters such as pulse amplitude [10], pulse rate (H-FIRE) [11], electrode arrays with a number of electrodes [12], and tissue conductivity [13], all of which influence the range and depth of IRE. When electrical energy is transferred to tissues, the parameter most affected by the change in the magnitude of the imposed voltage should be prioritized [14].

Current oncological therapies aim to induce apoptosis, a programmed cell death process involving caspase protein activation and termination, in tumor cells that exhibit aberrant cell cycle behavior [15]. Activating innate death mechanisms such as apoptosis is a milestone in cancer cell elimination and plays a major role in preventing the proliferation of unregulated cancer cells [16]. In contrast, IRE exposure can lead to necrosis, resulting in localized inflammation, cell lysis, and injury to surrounding cells. Thus, the pathologic outcome resulting from IRE is a combination of both apoptosis and necrosis. These differences in cell death are crucial for clinical efficacy because enhancing apoptosis through oncological treatments is a valid therapeutic strategy [17]. This strategy is supported by the fact that apoptosis induces minimal inflammation and damage to surrounding tissues, while necrosis is associated with increased inflammation and release of harmful cellular contents, promoting tumor growth and metastasis [18,19]. 

The cornerstone of contemporary anticancer therapy resides in immunogenic cancer cell death (ICD). This involves the sustained exposure of damage-associated molecular patterns (DAMPs) to the immune system [20]. Although apoptotic cell death has been traditionally regarded as having poor immunogenicity compared to necrotic cell death, it also releases DAMPs (e.g., ATP in the early phase and HMGB1 in the late phase) and displays immunostimulatory effects [20]. IRE is recognized as one of the inducers of ICD [21]. IRE induces a local antitumor immune response and remodels the tumor microenvironment. Locoregional therapy, including IRE, exposes cancer antigens, which are then processed by APCs for antigen presentation to naïve T cells [22]. As a result, tumor-specific cytotoxic T cells are activated and proliferate, migrating to both the tumor and distant lesions to destroy tumor cells, demonstrating an abscopal effect [22].

Although considerable progress has been made in understanding the clinical efficacy of the ablation of solid organs, data on the effect of IRE intensity on gastric tissue injury are limited. The gastrointestinal tract, including the stomach tissue, which is a hollow viscus, is favored for ablation therapy due to its thin, movable tissue that is easy to perforate, unlike that of solid organs. Both gastrointestinal endoscopists and interventionists should be cautious of the risks of stomach perforation, which can lead to disastrous outcomes such as emergent surgery following ablation therapy. Additionally, clinicians are expected to predict recovery, plan follow-up visits, and establish treatment schedules. Thus, this study focused on the damage patterns of gastric tissue using specific electrodes to establish standards of safety for mitigating tissue perforation, and understanding the extent to which changes in the intensity of electrical energy can impact the efficacy of inducing apoptosis as assessed by cleaved caspase-3 and terminal deoxynucleotidyl transferase nick-end labeling (TUNEL) in normal tissues. Furthermore, the gastric epithelial regeneration patterns in response to electrical mucosal damage were explored to predict the patterns and duration of recovery following IRE.

## 2. Materials and Methods

### 2.1. Ethical Statement

The Institutional Animal Care and Use Committee of the Korea University College of Medicine approved all research involving animals (approval number: KOREA-2019-0079). Data acquisition, analysis, and interpretation were performed in accordance with the Animal Research Reporting of In Vivo Experimental Guidelines. 

### 2.2. Study Design

**Figure 1:**
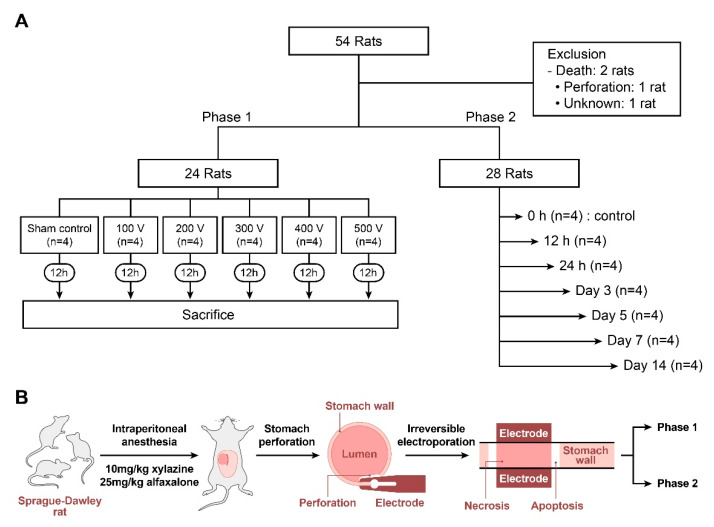
Schematic illustration of rat experiment. (**A**) Flowchart of the experimental design. (**B**) A method of establishing a rat irreversible electroporation (IRE) model.

This study consisted of two phases. Phase 1 involved assessing gastric mucosal damage, including apoptosis and necrosis, in response to electrical energy. Phase 2 was designed to explore the gastric epithelial regeneration process and the extent of fibrosis within a given time (Figure 1A). The experimental group consisted of 52 rats arbitrarily divided into two phases. Twenty-four rats were assigned to phase 1 and divided into six groups exposed to different voltages—the unmanipulated sham control group (*n* = 4) that underwent abdominal surgery without IRE; the 100 V group (*n* = 4) that underwent abdominal surgery with 100 V-IRE; the 200 V group (*n* = 4) that underwent abdominal surgery with 200 V-IRE; the 300 V group (*n* = 4) that underwent abdominal surgery with 300 V-IRE; the 400 V group (*n* = 4) that underwent abdominal surgery with 400 V-IRE; and the 500 V group (*n* = 4) that underwent abdominal surgery with 500 V-IRE. The phase 2 study included 28 rats for evaluation of mucosal regeneration. The rats underwent IRE stimulation at 400 V, and the number of collagen fibers was assessed at 12 h and on days 1, 3, 5, 7, and 14 after ablation. The 400 V used in the phase 2 study was determined based on the results of the phase 1 study, ensuring the safety of stomach tissue and observing the longest recovery period from maximal gastric damage.

### 2.3. IRE Rat Model

**Figure 2:**
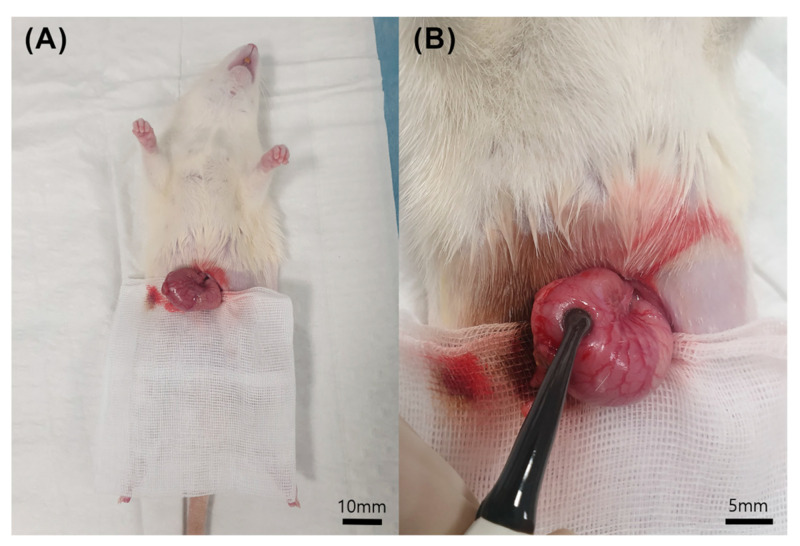
Irreversible electroporation animal model. (**A**) Rat model of the sham-operated control group for stomach irreversible electroporation (IRE); (**B**) IRE procedure on the stomach (electrode diameter, 3 mm).

Female Sprague–Dawley rats (aged seven weeks, weighing 190–210 g) were used for all experiments (Figure 1B). The rats were allowed to acclimatize to laboratory conditions for seven days and fasted for 24 h before the experiment. Subsequently, the rats were anesthetized by an intraperitoneal injection of 10 mg/kg xylazine and 25 mg/kg alfaxalone [23]. After 30 min, the hair over the abdominal area was shaved, and the skin was disinfected with alcohol before being incised. Rats were injected subcutaneously with 5 mg/kg ketoprofen, followed by 5 mg/kg enrofloxacin [24]. A midline abdominal incision was made from the sternum towards the umbilicus to expose the stomach, measuring approximately 2 cm [25]. Subsequently, a 0.5 cm incision was made in the greater curvature of the stomach for IRE electrode access (Figure 2). Intragastric contents were irrigated with normal saline (5 mL). Electroporation was performed on the mucosal surface of the stomach, resulting in the creation of four lesions in the antrum and body. The incised stomach was closed using sutures. Finally, the abdominal wall was closed after irrigating the intraperitoneal space with 5 mL of normal saline. The sham control group underwent the same procedure but without IRE ablation.

### 2.4. IRE Protocol and Modeling

**Figure 3:**
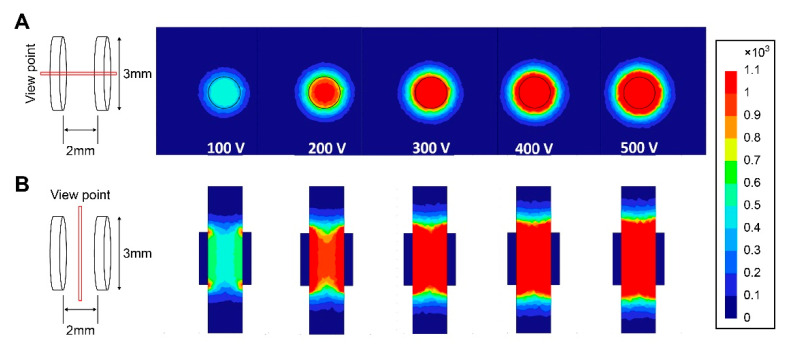
Simulation of varying electric field intensity for irreversible electroporation depending on the voltage. (**A**) in a parallel plane of wave propagation, (**B**) in a vertical plane of wave propagation; red indicates high electrical field intensity and blue indicates low electrical field intensity (scale bar unit, V/cm).

IRE was performed using BTX Gemini X2 (Harvard Apparatus, Holliston, MA, USA) and Tweezerodes (Harvard Apparatus). The electrode consisted of a round platinum plate (diameter, 3 mm). The electrodes were placed 2 mm apart to electrically stimulate the gastric tissues. The electrical stimulation conditions used were as follows: pulse interval of 0.1 s, pulse length of 0.1 ms, and 40 total administered pulses. To adjust the IRE energy, the pulse amplitude was gradually increased from 100 to 500 V (at 100 V intervals), corresponding to an increase in the electrical field intensity from 500 to 2500 V/cm. The electrical field based on the electrode and electrical parameters was simulated and modeled using the COMSOL Multiphysics 5.6b software (COMSOL Inc., Stockholm, Sweden) (Figure 3) [26].

### 2.5. Hematoxylin and Eosin (H&E) Staining

Rats were euthanized in a CO_2_ chamber for 10 min. The previously sutured abdomen was opened, and the resected stomach was incised at the greater curvature. Gastric tissues were fixed in a solution of 10% formaldehyde for 24 h before being embedded in paraffin blocks and sliced into 3 μm thick sections. H&E-stained slides were examined under a light microscope (BX51; Olympus, Tokyo, Japan).

### 2.6. TUNEL Assay

TUNEL staining was performed to detect DNA fragmentation using an in situ cell death detection kit (ApopTag Kit-S7100; Millipore Sigma, Burlington, MA, USA) according to the manufacturer’s instructions. Sections deparaffinized with xylene were sequentially dehydrated in decreasing ethanol concentrations, followed by immersion in a 0.3% H_2_O_2_ solution for 15 min to inactivate endogenous peroxidase. Subsequently, formalin-fixed paraffin-embedded sections were incubated with proteinase K for 15 min to digest nucleases before being washed with phosphate-buffered saline and incubated with a terminal deoxynucleotidyl transferase enzyme solution for 1 h at 37 °C. The sections were incubated with an anti-digoxigenin antibody conjugate for 30 min at 37 °C and then stained with diaminobenzidine chromogen to detect peroxidase activity. Normal tonsil tissue was used as a positive control. A TUNEL-positive finding was defined as a case in which the nucleus and cytoplasm stained brown.

### 2.7. Immunohistochemistry (IHC) of Cleaved Caspase-3

Formalin-fixed, paraffin-embedded tissue blocks were used for IHC. Deparaffinized sections were rinsed twice with phosphate-buffered saline, and antigen retrieval was performed for 30 min in a microwave using a solution containing Tris (10 mM), EDTA (1 mM), and Tween 20 (0.03%). Endogenous peroxidases were inactivated using H_2_O_2_ (0.3%) for 15 min. The specimens were incubated with a primary rabbit polyclonal antibody (#9664, ASP 175, Cell Signaling Technology, Danvers, MA, USA) against cleaved caspase-3 (1:1000) for 1 h at 37 °C, followed by diaminobenzidine staining using the EnVision Detection System (K5007; DAKO, CA, USA). Control sections were stained with Mayer’s hematoxylin. Cleaved caspase-3 staining was considered positive when the background was clear, and the cytoplasm stained brown.

### 2.8. Masson Trichrome (MT) Staining

Tissue blocks were deparaffinized by immersion in three graded absolute xylene solutions and sequentially in a series of ethanol solutions. Sections were submerged in Bouin’s solution at 60 °C for 30 min and washed with running tap water for 2 min. The sections were stained with modified Weigert’s hematoxylin for 8 min to differentiate the nuclei and again washed under running water for 5 min. To stain the acidophilic cytoplasm of the muscle and collagen, the specimens were stained with the anionic dye Biebrich scarlet-acid fuchsin solution before washing with running distilled water for 2 min. After incubation with a phosphomolybdic–phosphotungstic acid solution for 6 min to selectively decolorize the collagen fibers, the sections were submerged in an aniline blue solution for 5 min to stain the fibroblasts and collagen, followed by washing. Subsequently, the specimens were decolorized using a 1% acetic acid solution and rinsed with distilled water. The sections were immersed in a series of graded alcohol solutions (once in 70% and 80% ethanol and twice in 95% and 100% ethanol) before being incubated with xylene (three times). The mounted specimens were covered with coverslip using Tissue-Tek Glas (Sakura, Torrance, CA, USA).

### 2.9. Digital Image Analysis

The samples were subjected to TUNEL, IHC, and MT staining and examined under an optical microscope (DX 51-T, Olympus, Tokyo, Japan). The amount of collagen in MT-stained, TUNEL-positive, and cleaved caspase-3 positive sections was analyzed using ImageJ 1.52a software (National Institutes of Health, Bethesda, MD, USA). Because the acquired images were polychromatic, a color deconvolution plugin was used for stained image separation. We used pre-existing hematoxylin and diaminobenzidine built-in vectors for TUNEL and cleaved caspase-3 staining and defined the vectors for MT-stained image separation analysis. Following the “FROM ROI” selection, we drew the three regions of interest over the most prominent staining area for better separation. After completing color deconvolution, threshold levels and regions of interest were selected to measure the lesion area [27]. Subsequently, TUNEL- and caspase-3-stained areas were calculated. 

### 2.10. Apoptosis, Necrosis, Apoptosis/NECROSIS Ratio Definition

In the H&E image, cytoplasmic condensation (pyknotic nucleus), loss of cell–cell contact, and cell shrinkage (apoptotic bodies) were defined as apoptosis, while cell swelling and rupture with associated surrounding tissue damage were defined as necrosis [28,29].

Positive TUNEL staining is generally considered indicative of cell death [30,31]. Apoptosis and necrosis were distinguished by cleaved caspase-3 staining positivity [29]. Thus, TUNEL-positive/cleaved caspase-3-positive areas (TUNEL+/Casp+) were considered apoptotic, while TUNEL-positive/cleaved caspase-3-negative areas (TUNEL+/Casp-) were considered necrotic [32]. The necrotic area was defined by subtracting the cleaved caspase-3-positive area (TUNEL+/Casp+) from the TUNEL-positive area. The apoptosis/necrosis ratio was determined using cleaved caspase-3 and TUNEL-positive areas to identify predominant cell death [33,34]. The apoptosis/necrosis ratio values were calculated by dividing the area of apoptosis by the area of necrosis. We ultimately obtained the apoptosis/necrosis ratio by dividing each ratio value by the value of the sham control group.

### 2.11. Statistical Analysis

All statistical analyses were performed using SPSS (version 24.0; SPSS Inc., Chicago, IL, USA). The Shapiro–Wilk test was used for normally distributed variables. Pearson’s correlation coefficients were calculated. Differences in the ablated area according to voltage and changes in the ablated area over time, TUNEL-positive areas, cleaved caspase-3-positive areas, apoptosis/necrosis ratio, and MT-positive areas among the groups were analyzed using analysis of variance (ANOVA) followed by Tukey’s post hoc test. Statistical significance was set at *p* < 0.05. All relevant data are expressed as the mean and standard deviation.

To calculate the minimum sample size, we used the F-test in G*Power (ver. 3.1.9.7, Heinrich-Heine-Universität Düsseldorf, Düsseldorf, Germany) for the ANOVA statistics of six groups. By setting the power to 0.8 and the number of each group to four, the computed effect size was 0.978 by incorporating the mean area of each group. Based on this calculation, the required number of animals for the experiment was 24, with a minimum of four animals per group. 

## 3. Results

### 3.1. Phase 1 Study

#### 3.1.1. Gross Appearance of the Ablated Stomach

**Figure 4:**
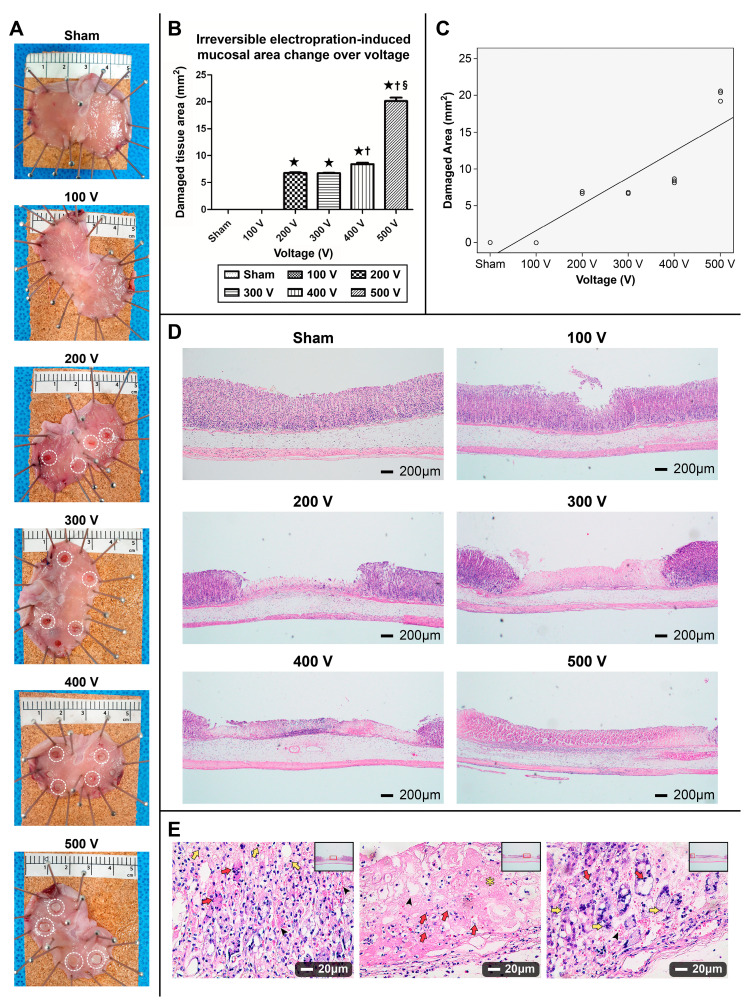
Gross tissue images with hematoxylin and eosin (H&E) staining after irreversible electroporation treatment in a rat model. (**A**) Photographs showing the condition of gastric tissue after irreversible electroporation at different electrical voltages (**B**) Irreversible electroporation (IRE) ablation of gastric tissue and effect of different electrical voltages on the area of gastric mucosal damage following IRE treatment in rats examined at the 12 h surviving time point (★ *p* < 0.01 vs. sham control and 100V group, ^†^ *p* < 0.01 vs. 200 V and 300 V group, ^§^ *p* < 0.01 vs. 400 V group). (**C**) Correlation between IRE-induced damaged area and electrical voltages (Pearson’s correlation coefficient 0.911, *p* < 0.001). (**D**) H&E staining of the electrical field intensity at regular intervals of voltage (40× magnification). (**E**) H&E staining for comparison between 200 and 400 V electroporation (400× magnification). The ablated mucosal center with 200 V electroporation showed pyknosis (yellow arrows), nuclear fragments, and protein aggregation from dead cells (red arrows and black arrows) (left), 400 V-ablated tissue center revealed cellular loss (black arrow), pinkish necrotic materials (yellow asterix), and fragmented cell debris (red arrows) (middle), and non-electroporated margins indicate numerous mixes of apoptotic and necrotic cells (protein aggregation: black arrowheads, pyknotic cells: red arrows, necrotic cell: yellow arrows) (right).

Electroporation at 100 V (500 V/cm) failed to induce mucosal damage, and tissue was similar to that observed in the sham control group (Figure 4A). At 200 V (1000 V/cm), electroporation resulted in a marginally depressed erythematous gastric erosion. The appearance of the electroporated mucosal layer at 300 V (1500 V/cm) was similar to that observed at 200 V. At 400 V (2000 V/cm), the ablated mucosa exhibited whitish mucosal changes in the ablated center and ring-shaped erythematous erosions at the ablated boundary. Gross inspection of the 500 V (2500 V/cm)-electroporated mucosa revealed a much larger ablated area than that of the 400 V-electroporated mucosa. 

#### 3.1.2. Damaged Gastric Tissue Area Varying by Electrical Field Intensity and Correlation between Area of the Ablation Zone and Electrical Voltage

No significant differences in the areas of damaged tissue were observed between the 200 and 300 V groups (6.79 mm^2^ vs. 6.74 mm^2^). The treated area at 400 (8.43 mm^2^) or 500 V (20.14 mm^2^) was significantly larger than the electroporated area at 200 or 300 V (*p* < 0.05). The ablated mucosal area at 500 V was almost three times larger than that at 200–300 V, (*p* < 0.01) (Figure 4B). A strong positive linear correlation between the damaged tissue area and the different voltages (Figure 4C).

#### 3.1.3. H&E Staining

H&E staining of tissues electroporated at 100 V tissues showed no histopathological differences compared to the sham control (Figure 4D). However, the center of the superficial mucosa electroporated at 200 V (1000 V/cm) showed minor depressions and necrotic changes. The mucosa electroporated at 200 V exhibited a loss of mucosal epithelial cells with inflammatory cell infiltration, and the layers of the structures were partially preserved. All the mucosal layers electroporated at 300 V exhibited necrosis and a clear demarcation between the electroporated and non-electroporated lesions was observed. Examination of the ablation zone at 400 V revealed preserved structural layers and reduced H&E staining due to lysis compared with the adjacent normal tissue. Both the 400 and 500 V-ablated groups exhibited necrotic expansion. Compared with the 200 V-ablated mucosa presenting the central ablation zone with pyknosis, the 400 V-ablated mucosa exhibited different apoptosis and necrosis patterns, with central necrosis and apoptosis of the ablated margin (Figure 4E).

#### 3.1.4. TUNEL Assay

**Figure 5:**
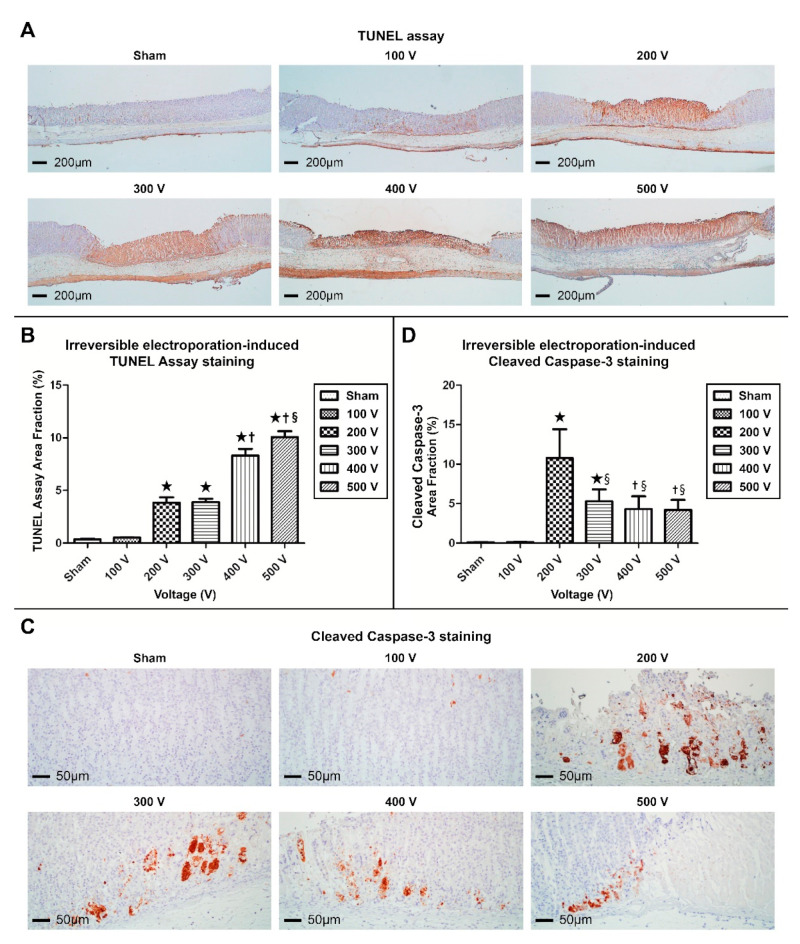
Terminal deoxynucleotidyl transferase dUTP nick-end labeling (TUNEL) and immunohistochemistry of anti-cleaved caspase-3 antibodies change depending on electric field distribution. (**A**) TUNEL staining of the electroporated gastric tissue (40× magnification). (**B**) Augmented activity of the TUNEL staining on the dose-dependent voltage (★ *p* < 0.01 vs. sham control and 100V group, ^†^ *p* < 0.01 vs. 200 and 300 V group, ^§^ *p* < 0.01 vs. 400 V group). (**C**) Immunohistochemical staining for cleaved caspase-3 in the ablated gastric tissue (200× magnification). (**D**) Biphasic activity of the cleaved caspase-3 levels on the dose-dependent voltage (★ *p* < 0.01 and ^†^ *p* < 0.05 vs. sham control and 100V group, ^§^ *p* < 0.01 vs. 300–500 V group).

In both the sham control and 100 V-electroporated gastric tissue groups, no brown nuclei or cytoplasm in the TUNEL assay (Figure 5A). The mucosal layers electroporated with 200 V displayed strong superficial mucosal gland staining and deficient staining at the base of the gastric gland. Only the submucosa and muscularis propria of the gastric tissue layers retained their original architecture. At 300 to 500 V, the gastric mucosa revealed evenly brown-stained nuclei and cytoplasm in all layers, and the boundary between the ablated and non-ablated areas was sharply demarcated.

The fraction of TUNEL-positive areas increased with electrical field strength (Figure 5B). Mucosae electroporated at 200 (3.83%) and 300 V (3.89%) exhibited significantly larger TUNEL-positive areas than those in the control group. However, no significant difference in the TUNEL-positive area between the 200 and 300 V-electroporated mucosae was observed. Additionally, the TUNEL-positive areas in the mucosa electroporated at 400 (8.32%) or 500 V (10.07%) were significantly larger than those in the mucosa electroporated at 200 or 300 V. Considering the size of the measured ablated area, the mucosa electroporated at 500 V exhibited the largest fraction of TUNEL-positive areas.

#### 3.1.5. Cleaved Caspase-3 Immunohistochemistry

IHC analysis of cleaved caspase-3 in the control group revealed the cytoplasmic staining of apoptotic superficial glandular cells (Figure 5C). The mucosa electroporated with 100 V showed similar cleaved caspase-3 staining results as the control group. The mucosal area electroporated at 200 V showed positive cytoplasmic and nuclear staining in the deep glandular layer of the ablation center. Furthermore, 300–500 V-ablated mucosae showed nuclear and cytoplasmic cleaved caspase-3-positivity at the margins of the deep mucosal layers.

Digital image analysis revealed that the proportion of cleaved caspase-3-positive areas was the largest in the 200 V-electroporated group (10.77%). Decreased expression levels of cleaved caspase-3 was observed after electroporation with voltages greater than 200 V. Although the fractions of the 300- (5.32%), 400- (4.57%), and 500 V (4.27%)-ablated groups were significantly higher than those of the 100 V–electroporated (0.13%) and control groups (0.10%), positive expression levels continuously decreased from 300 to 500 V, indicating that there is an inverse relationship with increases in electrical field strength (Figure 5D). 

#### 3.1.6. Staining Pattern upon Electrical Energy Intensity

**Figure 6:**
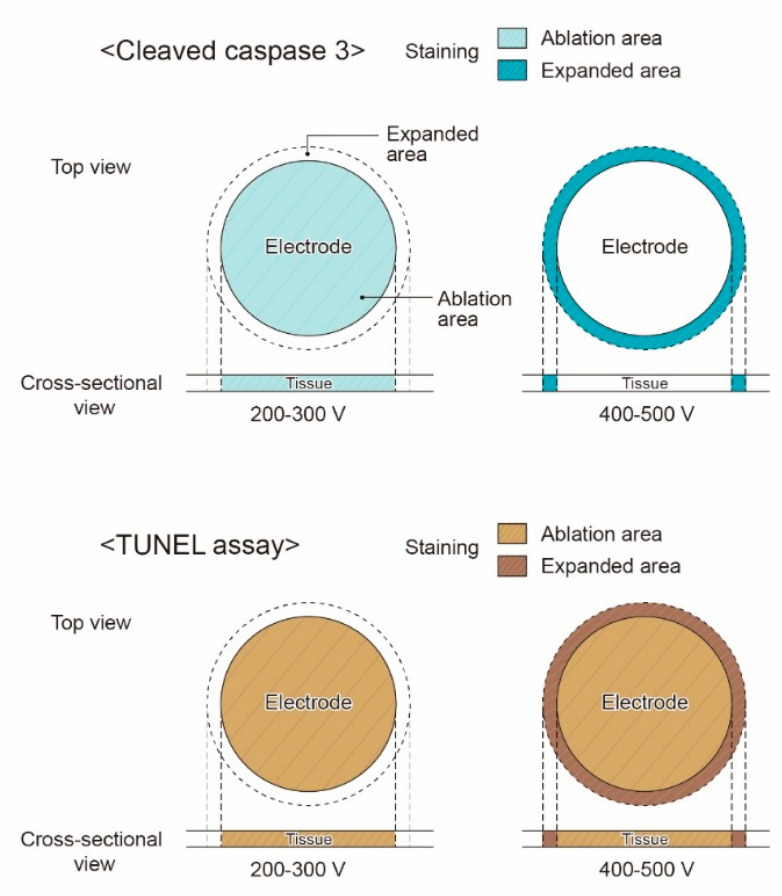
Illustration of cleaved caspase-3 and TUNEL staining pattern. TUNEL staining of the ablation zone in the central zone and ablation margins, whereas cleaved caspase-3 staining was expressed commonly at the ablation margin rather than at the center of the ablation zone, as the voltage increased from 200 to 300 and 400–500 V.

The cleaved caspase-3 IHC staining pattern differed from that observed in the TUNEL assay. In gastric tissue, IRE ablation results in two main stained areas: the central ablation area, forming a disc where the electrode contacts, and the marginal expanded area, staining in a ring shape at the margin where direct electrode contact is absent. The expanded area, where cleaved caspase-3 stains in a ring shape, indicates ring-shaped apoptosis under an electrical field threshold above a certain level. At voltages beyond 400–500 V, the expanded area manifests as a ring-shaped cleaved caspase-3–stained region surrounding the central ablation area. The cleaved caspase-3-stained area of the lamina propria transitioned from the ablation center to the margin, gradually decreasing with increasing electrical field strength (Figure 6). However, the TUNEL-positive area increased laterally and extended vertically from the superficial mucosal layer to the submucosa as electrical energy intensity increased. 

#### 3.1.7. Apoptosis/Necrosis (TUNEL+/Casp+ over TUNEL+/Casp-) Ratio

**Figure 7:**
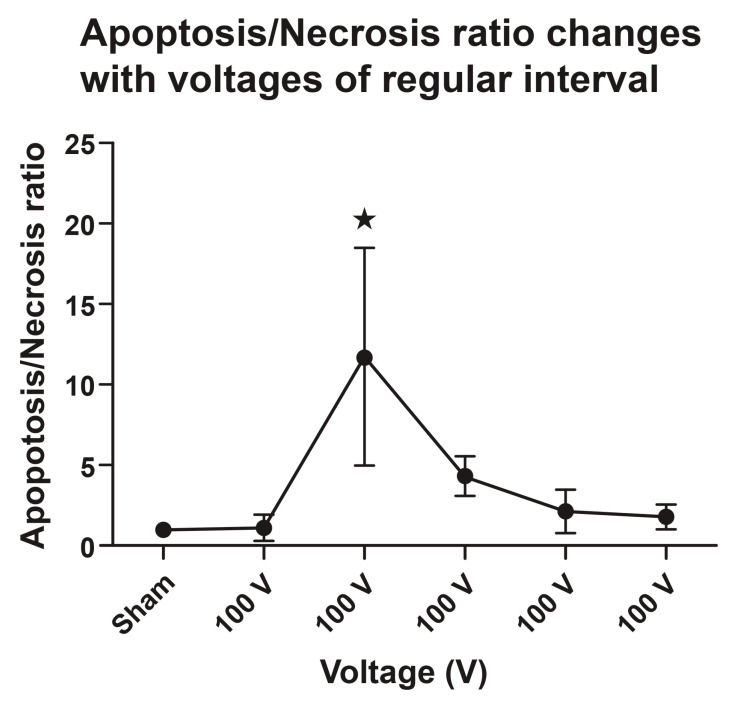
Apoptosis/necrosis ratio after IRE treatment and dose-dependent voltage. Increasing the voltage induces apoptosis/necrosis ratio changes in the gastric tissue through a biphasic cleaved caspase-3-dependent mechanism. One-way analysis of variance (ANOVA) was performed, followed by Tukey’s post hoc test. Data are expressed as mean and standard deviation (SD) (*n* = 4), ★ *p* < 0.01.

The ratio of the cleaved caspase-3-stained area to the TUNEL-stained area at the ablation site was calculated. The 200 V-electroporated gastric mucosae showed the highest ratio of 11.76, followed by the 300-, 400-, and 500 V-electroporated gastric mucosae at 4.35, 2.17, and 1.80, respectively (Table 1 and Figure 7).

### 3.2. Phase 2 Study

#### 3.2.1. Gross Appearance of Recovery in the Ablated Zone

**Figure 8:**
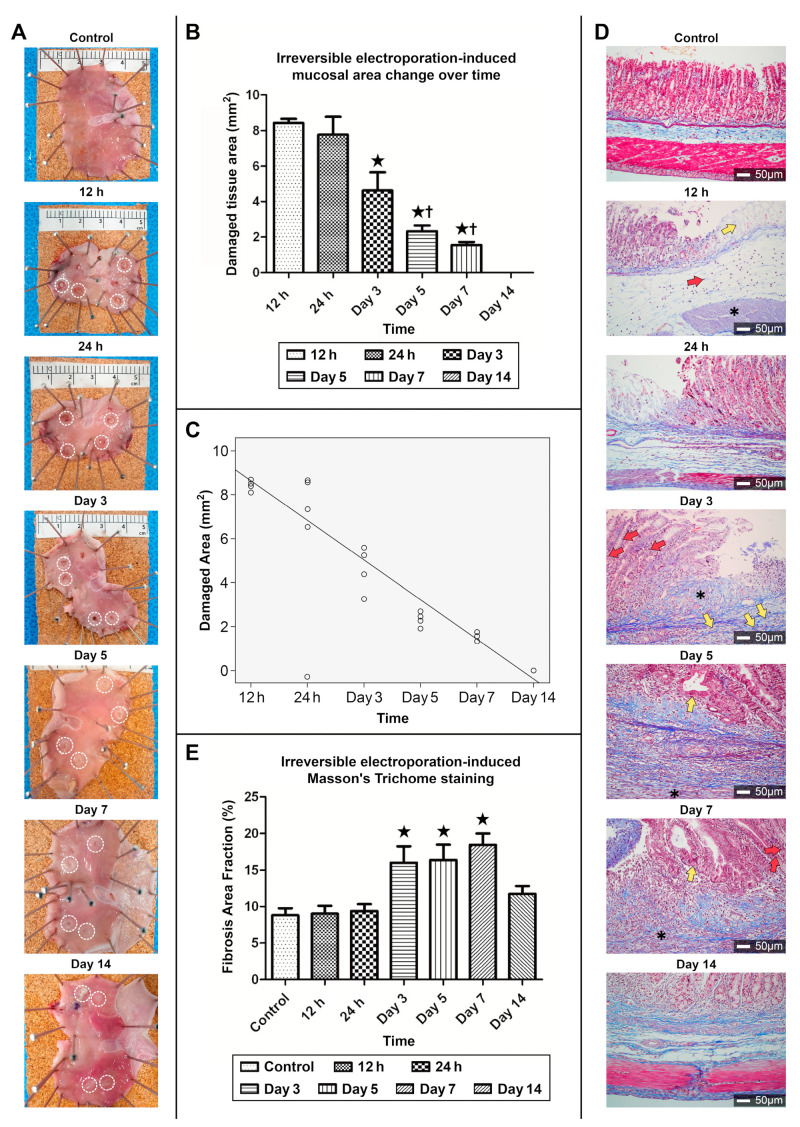
Changes with Masson Trichrome-stained histologic sections of gastric tissue recovery over time after ablation at 400 V. (**A**) Healing of electroporated gastric tissue examined for 14 days (white dot circle, IRE lesions). (**B**) Effects of 400 V (with 40 pulses, duration of 4 s) electrical voltage on gastric mucosal regeneration following irreversible electroporation (IRE)-induced injury in rats surviving at various time points. A decrease in the damaged gastric mucosal area was measured over time. Significance is represented as ★ *p* < 0.01 vs. 12 and 24 h group, ^†^ *p* < 0.01 vs. day 3 group. (**C**) Correlation between IRE-induced damaged area and time (Pearson correlation coefficient, 0.968, *p* < 0.001). (**D**) Masson trichrome staining of gastric tissues (12 h: lysed villi, yellow arrows; mononuclear cell infiltration, red arrows; lysed submucosa, asterisk, Day 3: mitotic epithelium, red arrows; collagen fibers, asterisk; immature fibroblasts, yellow arrows, Day 5: immature epithelium, yellow arrows; immature fibroblasts, asterisk, Day 7: immature epithelium, yellow arrows; mitotic figuration, red arrows; immature fibroblasts, asterisk, 200× magnification). (**E**) Sequential time change in the fibrosis area fraction on the IRE-induced ablation zone (★ *p* < 0.01 vs. control, 12 and 24 h, day 14 group).

At the initial follow-up examination after 12 h of ablation, the 400 V ablative zones exhibited a round, whitish mucosa with a surrounding hyperemic rim (Figure 8A). After 24 h of ablation, IRE lesions typically demonstrated devitalized necrotic mucosa, called slough, at the ablation center, similar to that observed 12 h after ablation. Three days after ablation, the peripheral enhanced ring enveloping the necrotic mucosa disappeared, with the advent of sharp, deep gastric ulcers. White fibrous tissue covering the shallow gastric ulcer margin first appeared five days after ablation. The IRE ablation zones after seven days showed a slight reduction in ulcer size and an increased density of surrounding white fibrous connective tissue. Fourteen days after IRE, the appearance of the ablative zones revealed invisible ulcer craters with marginal fibrous gastric tissue remaining without ulcer contraction or hypertrophic scars.

#### 3.2.2. Correlation between the Area of the Ablation Zone and Time

No significant differences were observed in the sizes of ablation-induced ulcers until 24 h post-ablation (8.43 mm^2^ vs. 7.77 mm^2^, *p* = 0.658) (Figure 8B). The size of the post-ablation ulcers gradually decreased from days 1 to 5. The areas of the ablation zones on days 5 and 7 were not significant (2.33 mm^2^ vs. 1.55 mm^2^, *p* = 0.494). On day 7, the most attenuated size of IRE-induced gastric ulcers was observed in the ablation zone (correlation coefficient 0.968, *p* < 0.001) (Figure 8C).

#### 3.2.3. MT Staining of the Collagen Fibers in the Ablation Zone

The mucosa of the control group exhibited well-organized normal glands with visible chief, parietal, and mucous neck cells (Figure 8D). The mucosal layers were lysed 12 h post-ablation and mononuclear immune cells infiltrated the submucosa. After 24 h, a greater number of inflammatory cells had infiltrated the mucosa and submucosa in the ablation zone, and apoptotic cells appeared at the margins. No significant difference in the tissue structure or amount of collagen fibers 24 h post-ablation was observed. On day 3 post-ablation, neutrophils and acute inflammatory cells were recruited to the ablation zone, and mitotic activity of epithelial cells, indicative of proliferation and regeneration, was first observed in the ablated margin. The ablation margin consisted of markedly enhanced collagen fibers surrounded by the extracellular matrix. An increased number of immature fibroblasts were distributed throughout the muscularis propria. Starting from day 5 post-ablation, the proliferation of mitotic gastric epithelium with stromal fibroblasts at the ablation margin increased compared with that on day 3 post-ablation. Proliferated collagen fiber accumulation was extensively present in the IRE ablation zone, which covered the lamina propria of the ablation center. Despite the progression of mucosal regeneration, the recovered gastric glands showed architectural distortion and disorganization due to the presence of immature mucosal epithelium. Disorganized immature epithelial cells at the ablation center completely filled the mucosal defect with increased collagen fibers on day 14. Although the mucosa recovery was incomplete, epithelial cell-structured glands and a villous architecture were observed.

#### 3.2.4. Fibrosis Area Fraction Changes in MT Staining upon Ulcer Healing

Compared with the control group (8.8%), MT staining of fibrous tissue in the ablation zone at 12 h (9.04%) and 24 h (9.41%) post-ablation revealed non-enhanced collagen formation (Figure 8E). Although mucosal and submucosal fibrosis increased significantly from day 3 (16.0%) to day 7 (18.44%) post-ablation compared with the 24 h ablated tissue, no significant differences in collagen concentrations were observed between days 3 and 7. The amount of fibrous regenerated collagen on day 14 decreased compared with that on day 7 and was similar to the amount of collagen on day 1.

## 4. Discussion

Gastric cancer is the fifth most common malignancy and the fourth leading cause of cancer-related mortality worldwide, posing a major socioeconomic burden [35]. In Republic of Korea, gastric cancer ranks fifth (21.7) in terms of age-standardized incidence (per 100,000) among all cancer types [36], and patients with metastatic gastric cancer undergoing palliative chemotherapy have a poor prognosis [37]. Although endoscopic submucosal dissection (ESD) is primarily used for treating early gastric cancer without lymph node metastasis, surgical resection is considered the standard treatment for locally advanced metastatic gastric cancer with positive lymph nodes. Current ESD and gastrectomy face challenges with clinically unmet patient needs, such as large superficial early gastric cancer in older patients [38], expanded indications for ESD [39], proximal advanced gastric cancer [40], and partial gastric outlet obstruction [41]. Current guidelines for these issues remain controversial and inconclusive; hence, apoptosis induction via non-thermal IRE ablation can be considered an attractive alternative because it triggers immunogenic cascades that debulk and selectively ablate tumors and has a relatively short procedure time compared with ESD and surgery.

Our investigation demonstrated biphasic activation of gastric IRE-induced apoptosis in response to the electrical field intensity. Biphasic activation comprises phase 1, characterized by an increase in the apoptosis/necrosis ratio up to 200 V, and phase 2, with a subsequent decrease up to 500 V. The main research findings highlighted the most efficient electrical energy, indicating that the highest apoptosis/necrosis ratio (1000 V/cm) and IRE treatment-induced ulceration resulted in extracellular matrix (ECM) recovery within two weeks. Although TUNEL reveals greater mucosal damage with increasing electrical field intensity, the cleaved caspase-3-positive staining fraction gradually decreased after 1000 V/cm, the point at which maximum damage was created by the electrical intensity. When the electric field strength exceeds a specific critical point, necrosis rather than apoptosis becomes the dominant mechanism of cell death. These findings suggest that applying a specific amount of energy can safely promote gastric mucosal ablation and maintain gastric architecture and function.

IRE has been continuously used for gastric mucosal ablation. Li et al. attempted IRE ablation of the stomach-serosal layer in Tibetan minipigs, followed by histological safety evaluations [42]. Lee et al. demonstrated the applicability of IRE in targeting mucosal lesions in the stomachs of rats [43]. One study performed IRE using a magnetic anchoring electrode on the rabbit stomach wall via an open surgical approach to confirm its safety [44]. Another study confirmed the feasibility of endoscopic IRE for mucosal ablation in the beagle stomach using magnetic anchoring and guidance-assisted techniques [45]. Jeon et al. confirmed the feasibility and effectiveness of minimally invasive IRE using an endoscopic needle-type IRE catheter [46]. Furthermore, Zhang et al. demonstrated that stomach physiology was preserved and histology was recovered after IRE in the rat stomach [25].

TUNEL staining was beneficial in predicting the area and depth of tissue damage in the mucosal layer of the electroporated areas according to the IRE electrical field strength. Although the IRE results using 1000 V/cm lacked TUNEL staining in the deep mucous gland cells at the ablation center, electroporation with an electrical field strength above 1500 V/cm enhanced cell death in the mucosal glands, which is indicative of the regeneration and loss of gastric mucosal function. TUNEL staining of the nuclei and cytoplasm of IRE-stimulated cells by exposing DNA fragments within the cell nucleus to the cytoplasm after the nuclear membrane breakdown was consistent with previous findings [5]. The TUNEL results also suggested that the ablation area can be strengthened as the electrical energy increases, and the ablation depth can be controlled by adjusting the electrical energy intensity.

Apoptosis is a complex and interlinked process resulting from IRE-induced cell injury. Typically, the following events occur sequentially during cellular injury-induced apoptosis: membrane damage, DNA and protein damage, increased reactive oxygen species production, calcium influx into the cells, mitochondrial damage, and ATP depletion [2,6]. The apoptotic pathway occurs sequentially through the intrinsic caspase-dependent pathway, which involves the mitochondrial pathway, leading to the activation of caspases 3, 7, and 9 [47,48,49] and enhancement of cytochrome c and oxidase IV levels [50,51]. In addition, pro-apoptotic factors such as BAX, BAK, and BAD are activated, whereas anti-apoptotic factors such as Bcl-2 and Bcl-xL are inactivated [52,53,54].

Caspases of inactive zymogens are mediators of apoptosis and are activated by proteolytic cleavage. Cleaved caspase-3 amplifies downstream apoptotic signaling [55]. Therefore, cleaved caspase-3 is a valuable marker of apoptosis. Active-caspase-3 initially appears in the cytoplasm during the early stages of apoptosis and later in the nucleus [56]. In this study, cleaved caspase-3 staining was predominantly observed in viable cells after IRE stimulation. Antibodies against cleaved caspase-3 produced irregular cytoplasmic staining of the superficial mucosal glands at the ablation center below 1000 V/cm and in deep mucosal glands at the ablation margin above 1500 V/cm. The location of cleaved caspase-3 IHC staining moved from the center of the electrodes to the margins as well as from the superficial to deep layers of the mucosa. Furthermore, staining intensity decreased with increasing electrical energy. IHC staining patterns allowed us to understand the biphasic activity of IRE-induced apoptosis. Upon reaching an electrical intensity of 1000 V/cm, apoptosis becomes the predominant mechanism, leading to an increase in the apoptosis/necrosis ratio; however, necrosis becomes the driving mechanism at levels beyond this threshold, leading to a decrease in the apoptosis/necrosis ratio. We demonstrated that the biological response (e.g., apoptosis) to linear increases in electrical field intensity does not follow a linear pattern but instead exhibits a biphasic pattern of an initial increase in apoptosis/necrosis ratio followed by a decrease. One plausible explanation for the staining pattern of cleaved caspase-3 is the time at which tissue specimens were obtained. Our phase 1 experiments detected apoptosis at various electrical energies at 12 h post-IRE. However, stimuli-induced apoptosis occurs at different times depending on the cell, tissue, and stimulation [57,58]. Further investigation of the time-dependent apoptotic changes is required.

We hypothesized that the efficacy of IRE in inducing apoptosis would differ depending on the intensity of the electrical field. In this study, we observed attenuated apoptosis and augmented necrosis with increasing electric field intensities. This can be explained by Joule heating, which refers to generating heat as current flows through a conductor. Theoretically, IRE is a nonthermal therapy. Some studies have reported that not all IRE conditions are nonthermal, as IRE involving high electrical field strength can induce thermal damage. Applying 2500 V/cm with 90 pulses to the liver increased the temperature above 50 °C, forming a white zone representative of typical coagulative necrosis [59]. In another study, when the prostate was electroporated with an electrical field strength calculated from the bio-heat equation, IRE-generated thermal energy increased the tissue temperature to approximately 67–92 °C [60]. Previous IRE studies of the liver revealed that the marked white zone after ablation corresponded to the area of thermal coagulation [61]. The highest level of apoptosis was observed at a relatively low electric field intensity of 1000 V/cm. A biphasic dose response was observed in a previous study that reported apoptosis as a mechanism of cell death after nanosecond-pulsed electrical field stimulation. This study reported that nanosecond pulsed electrical field stimulation of up to 40 kV/cm after 24 h activated caspase-3/7 in an electric field-dependent biphasic manner [50]. Our findings suggest that regulating the apoptosis/necrosis ratio of gastric tissues using IRE influences the tumor microenvironment and immune system.

Based on the findings of this study, the maximum electrical field strength required to ablate gastric tissues was 2500 V/cm (40 pulses). The ablated tissue exhibited a white zone with peripheral ring-shaped erythematous mucosal erosion at 2000 and 2500 V/cm. The cleaved caspase-3-positive area was compatible with the ring-shaped erosion. In particular, the simulated marginal electric field strength during the application of 2000 V/cm was 800–1200 V/cm, which correlated with our finding that apoptosis occurred most frequently at 1000 V/cm. The adverse events associated with an electrical field strength of 2500 V/cm resulted in the death of two rats following ablation at 2500 V/cm. The autopsy revealed that one rat died of stomach perforation, whereas the cause of death for the other was unclear. Therefore, for IRE in the stomach, it is advisable to use an electrical field strength of less than 2500 V/cm to avoid adverse events and increase the efficacy of the procedure in promoting apoptosis. Additionally, although the apoptosis rate decreased as the electrical energy exceeded the optimal electric field strength, employing 2000 V/cm may be beneficial in cases where a large tumor area needs to be ablated.

The total number of collagen fibers in the ablated area was measured to evaluate the regeneration of IRE-induced ulcers. At 24 h post-ablation, no significant difference in the number of collagen fibers was observed. However, a significant increase in profibrotic tissue was observed between 24 h and 3 days post-ablation, which is consistent with the results of previous studies [62]. Although attenuated regeneration of radiofrequency ablation-induced eliminated spaces has been documented even 10 weeks after ablation, IRE-induced mucosal defects recover visually and histologically within two weeks [63]. Compared with peptic ulcers, ESD-induced ulcers usually require 8–12 weeks for restoration [64]. Even after high electrical stimulation, the preserved ECM observed in our study could be attributed to enhanced recovery. These findings are consistent with previous studies that have reported the immunomodulatory role of the ECM [65]. Many studies investigating the functional role of the ECM have considered it an active source of immune-related molecules and passive structures [66]. The ECM proteins and the injured matrix directly modulate immune responses and release cytokines and growth factors that promote immune cell recruitment. Another hypothesis suggests that electrical stimulation promotes regeneration by activating stem cells in the gastric glands. The effects of electrical stimulation on cell migration, proliferation, and differentiation have been documented [67]. Therefore, in future studies, it is necessary to investigate whether the differentiation and healing of the gastric glands are related to stem cells.

Apoptosis, which is associated with the initiation of organ fibrosis and collagen formation and degradation, is upregulated at the site of wound healing [68]. Cytokines are secreted when macrophages phagocytose apoptotic cells, which results in the formation of profibrotic tissues. Therefore, the cell death mechanism of IRE may contribute to the faster recovery owing to abundant apoptosis, which results in fibrosis via preserved ECM, promoting immune cell recruitment and the release of cytokines, compared with other treatment modalities.

Considering the IRE parameters under electrical conditions, the electrical field strength and ablation time are key factors required for adjusting the electrical energy delivery. IRE develops when the electrical energy density exceeds a critical value and is accompanied by reversible electroporation, where Wc is the specific energy at which thermal ablation occurs [69,70]. The electrical energy density of IRE was calculated as follows:

(1) W=p·t

(2) $$p=\frac{E^{2}}{\rho }$$

(3) $$W=\frac{E^{2}\cdot t}{\rho }$$

where *W* is the electrical energy density, *p* is the electrical power, *t* is the ablation time, *E* is the electrical field, and ρis the tissue resistivity. The Wc energy of the stomach indicated that electroporation at 2000 V/cm was possible. According to Equation (3), thermal coagulation can be predicted to occur when the ablation time increases 4-fold for stimulations at 1000 V/cm and 1.78-fold for stimulations at 1500 V/cm. Based on this model, the appropriate electrical field strength and time to avoid thermal coagulation for gastric tissue ablation can be predicted.

The main strengths of our study are the tissue-level quantification of IRE-induced apoptosis and necrosis in the stomach based on IHC, and quantitatively determining an electrical field strength that leads to the best efficiency. Based on these findings, we could maximize the beneficial immunologic cascade effects of IRE at 1000 V/cm and minimize adverse events such as tremors, perforation, and pain. Another advantage of this investigation was the sequential observation of healing patterns in IRE-induced gastric ulcers. Our findings provide valuable information on the time-dependent proliferation, regeneration patterns, and extent of fibrosis in IRE-induced gastric ulcers.

This study has a few limitations. First, an electrical field was applied to normal tissue rather than tumor tissue. Owing to the differences in tissue components, gap junctions, cellular pathways, and gene expression patterns between cancerous and normal tissues, tumors exhibit abnormal responses to electrical stimuli [71]. Although the experiment was not conducted on gastric tumor tissue, we consider the findings valuable in terms of understanding the impacts of IRE on gastric tissue, for considerations over safety, therapeutic optimization, and further comparative research. Second, our study showed that IRE-induced ulcers at 2000 V/cm were able to undergo a rapid healing process. We assumed that this finding of accelerated regeneration could be attributed to the idea that the apoptosis level is associated with the electrical field intensity. Given that apoptosis and preserved ECM promote healing via immune cell activation, we demonstrated how healing periods and patterns of electrical intensity at 1000 V/cm differ from those at 2000 V/cm. This has important implications for understanding whether augmented apoptosis in tumors is linked to regeneration. Third, we determined the tissue apoptosis/necrosis ratio of electrical intensity using IHC staining. Although semi-quantitative IHC is a well-established method that allows the detection of protein expression, a direct quantification of activated proteins in apoptotic signaling pathways, including cleaved caspase-3 and BAX, would be better for confirming apoptotic protein levels and determining intrinsic or extrinsic apoptotic pathways. Finally, the number of experimental subjects per group in this study was relatively small. Increasing the participant quantity can enhance the statistical significance and reliability of the results.

## 5. Conclusions

This study highlighted the biphasic regulation of apoptosis and necrosis following IRE treatment with respect to electrical intensity. TUNEL and cleaved caspase-3 IHC showed that an electrical field strength of 1000 V/cm induced the highest apoptosis/necrosis ratio. The levels of collagen fiber deposition after IRE treatment continued to peak on day 7 post-ablation and decreased to their original levels on day 14. IRE has clinical applications owing to its therapeutic efficacy, as demonstrated by its ability to potentiate apoptosis and improve recovery. This implies that it is a suitable, minimally invasive procedure for next-generation ablation therapy in gastric cancer management.

## Figures and Tables

**Table 1:** Ratios of apoptosis over necrosis.

	Sham Control (*n* = 4)	100 V (*n* = 4)	200 V (*n* = 4)	300 V (*n* = 4)	400 V (*n* = 4)	500 V (*n* = 4)
TUNEL+/Casp+ over TUNEL+/Casp- ratio	1.00	0.455	10.8	3.29	1.42	0.959
	1.00	0.564	4.32	3.50	0.811	1.34
	1.00	2.27	20.7	5.90	2.74	2.29
	1.00	1.23	11.2	4.73	3.73	2.60

## Data Availability

All data generated or analyzed during this study are included in this published article.

## References

[1] Kotnik T., Rems L., Tarek M., Miklavčič D. (2019). Membrane Electroporation and Electropermeabilization: Mechanisms and Models. Annu. Rev. Biophys..

[2] Wasson E.M., Alinezhadbalalami N., Brock R.M., Allen I.C., Verbridge S.S., Davalos R.V. (2020). Understanding the role of calcium-mediated cell death in high-frequency irreversible electroporation. Bioelectrochemistry.

[3] Campana L.G., Edhemovic I., Soden D., Perrone A.M., Scarpa M., Campanacci L., Cemazar M., Valpione S., Miklavčič D., Mocellin S. (2019). Electrochemotherapy—Emerging applications technical advances, new indications, combined approaches, and multi-institutional collaboration. Eur. J. Surg. Oncol..

[4] Aycock K.N., Davalos R.V. (2019). Irreversible Electroporation: Background, Theory, and Review of Recent Developments in Clinical Oncology. Bioelectricity.

[5] Al-Sakere B., André F., Bernat C., Connault E., Opolon P., Davalos R.V., Rubinsky B., Mir L.M. (2007). Tumor ablation with irreversible electroporation. PLoS ONE.

[6] Batista Napotnik T., Polajžer T., Miklavčič D. (2021). Cell death due to electroporation—A review. Bioelectrochemistry.

[7] Rubinsky B., Onik G., Mikus P. (2007). Irreversible electroporation: A new ablation modality—Clinical implications. Technol. Cancer Res. Treat..

[8] Davalos R.V., Mir L.M., Rubinsky B. (2005). Tissue ablation with irreversible electroporation. Ann. Biomed. Eng..

[9] Edd J.F., Horowitz L., Dávalos R.F., Mir L.M., Rubinsky B. (2006). In vivo results of a new focal tissue ablation technique: Irreversible electroporation. IEEE Trans. Biomed. Eng..

[10] Geboers B., Scheffer H.J., Graybill P.M., Ruarus A.H., Nieuwenhuizen S., Puijk R.S., van den Tol P.M., Davalos R.V., Rubinsky B., de Gruijl T.D. (2020). High-Voltage Electrical Pulses in Oncology: Irreversible Electroporation, Electrochemotherapy, Gene Electrotransfer, Electrofusion, and Electroimmunotherapy. Radiology.

[11] Arena C.B., Sano M.B., Rossmeisl J.H., Caldwell J.L., Garcia P.A., Rylander M.N., Davalos R.V. (2011). High-frequency irreversible electroporation (H-FIRE) for non-thermal ablation without muscle contraction. Biomed. Eng. Online.

[12] Adeyanju O., Al-Angari H.M., Sahakian A.V. (2012). The optimization of needle electrode number and placement for irreversible electroporation of hepatocellular carcinoma. Radiol. Oncol..

[13] Golberg A., Bruinsma B.G., Uygun B.E., Yarmush M.L. (2015). Tissue heterogeneity in structure and conductivity contribute to cell survival during irreversible electroporation ablation by “electric field sinks”. Sci. Rep..

[14] Hogenes A.M., Overduin C.G., Slump C.H., van Laarhoven C.J.H.M., Fütterer J.J., Broek R.P.G.T., Stommel M.W.J. (2023). The Influence of Irreversible Electroporation Parameters on the Size of the Ablation Zone and Thermal Effects: A Systematic Review. Technol. Cancer Res. Treat..

[15] Carneiro B.A., El-Deiry W.S. (2020). Targeting apoptosis in cancer therapy. Nat. Rev. Clin. Oncol..

[16] Lopez J., Tait S.W.G. (2015). Mitochondrial apoptosis: Killing cancer using the enemy within. Br. J. Cancer.

[17] Ghobrial I.M., Witzig T.E., Adjei A.A. (2005). Targeting apoptosis pathways in cancer therapy. CA Cancer J. Clin..

[18] Yamamoto A., Huang Y., Krajina B.A., McBirney M., Doak A.E., Qu S., Wang C.L., Haffner M.C., Cheung K.J. (2023). Metastasis from the tumor interior and necrotic core formation are regulated by breast cancer-derived angiopoietin-like 7. Proc. Natl. Acad. Sci. USA.

[19] Liu Z.-G., Jiao D. (2020). Necroptosis, tumor necrosis and tumorigenesis. Cell Stress.

[20] Zhou J., Wang G., Chen Y., Wang H., Hua Y., Cai Z. (2019). Immunogenic cell death in cancer therapy: Present and emerging inducers. J. Cell. Mol. Med..

[21] He C., Huang X., Zhang Y., Lin X., Li S. (2020). T-cell activation and immune memory enhancement induced by irreversible electroporation in pancreatic cancer. Clin. Transl. Med..

[22] Xie L., Meng Z. (2023). Immunomodulatory effect of locoregional therapy in the tumor microenvironment. Mol. Ther..

[23] Siriarchavatana P., Ayers J.D., Kendall L.V. (2016). Anesthetic activity of alfaxalone compared with ketamine in mice. J. Am. Assoc. Lab. Anim. Sci..

[24] Flatow E.A., Komegae E.N., Fonseca M.T., Brito C.F., Musteata F.M., Antunes-Rodrigues J., Steiner A.A. (2017). Elucidating the role of leptin in systemic inflammation: A study targeting physiological leptin levels in rats and their macrophages. Am. J. Physiol. Regul. Integr. Comp. Physiol..

[25] Zhang Y., Han X., Li Z., Zhang Y., Liang L., Ma X., Liu H., Gao Y., Li Q., Chen X. (2021). Physiological and histopathological effects of electroporation pulse on stomach of rats. BMC Gastroenterol..

[26] Jeon H.J., Choi H.S., Lee J.M., Kim E.S., Keum B., Jeen Y.T., Lee H.S., Chun H.J., Jeong S., Kim H.B. (2023). Assessment of efficacy and safety of advanced endoscopic irreversible electroporation catheter in the esophagus. Sci. Rep..

[27] Crowe A.R., Yue W. (2019). Semi-quantitative determination of protein expression using immunohistochemistry staining and analysis: An integrated protocol. Bio-Protocol.

[28] Garrity M.M., Burgart L.J., Riehle D.L., Hill E.M., Sebo T.J., Witzig T. (2003). Identifying and Quantifying Apoptosis: Navigating Technical Pitfalls. Mod. Pathol..

[29] Kari S., Subramanian K., Altomonte I.A., Murugesan A., Yli-Harja O., Kandhavelu M. (2022). Programmed cell death detection methods: A systematic review and a categorical comparison. Apoptosis.

[30] Duan W.R., Garner D.S., Williams S.D., Funckes-Shippy C.L., Spath I.S., Blomme E.A. (2003). Comparison of immunohistochemistry for activated caspase-3 and cleaved cytokeratin 18 with the TUNEL method for quantification of apoptosis in histological sections of PC-3 subcutaneous xenografts. J. Pathol..

[31] Mirzayans R., Murray D. (2020). Do TUNEL and Other Apoptosis Assays Detect Cell Death in Preclinical Studies?. Int. J. Mol. Sci..

[32] Janke L.J., Ward J.M., Vogel P. (2019). Classification, Scoring, and Quantification of Cell Death in Tissue Sections. Vet. Pathol..

[33] Grusch M., Polgar D., Gfatter S., Leuhuber K., Huettenbrenner S., Leisser C., Fuhrmann G., Kassie F., Steinkellner H., Smid K. (2002). Maintenance of ATP favours apoptosis over necrosis triggered by benzamide riboside. Cell Death Differ..

[34] Mailleux A.A., Overholtzer M., Schmelzle T., Bouillet P., Strasser A., Brugge J.S. (2007). BIM regulates apoptosis during mammary ductal morphogenesis, and its absence reveals alternative cell death mechanisms. Dev. Cell.

[35] Sung H., Ferlay J., Siegel R.L., Laversanne M., Soerjomataram I., Jemal A., Bray F., Bsc M.F.B., Me J.F., Soerjomataram M.I. (2021). Global Cancer Statistics 2020: GLOBOCAN Estimates of Incidence and Mortality Worldwide for 36 Cancers in 185 Countries. CA Cancer J. Clin..

[36] Jung K.-W., Kang M.J., Park E.H., Yun E.H., Kim H.-J., Kong H.-J., Im J.-S., Seo H.G. (2023). Prediction of Cancer Incidence and Mortality in Korea, 2023. Cancer Res. Treat..

[37] Park S.H., Kang M.J., Yun E.H., Jung K.-W. (2022). Epidemiology of Gastric Cancer in Korea: Trends in Incidence and Survival Based on Korea Central Cancer Registry Data (1999–2019). J. Gastric Cancer.

[38] Chiu P.W.-Y., Sung J.J.-Y. (2011). Endoscopic resection for early gastric cancer: One piece is better than dash to pieces. Gastrointest. Endosc..

[39] Japanese Gastric Cancer Association (2017). Japanese gastric cancer treatment guidelines 2014 (ver. 4). Gastric Cancer.

[40] Li S., Gu L., Shen Z., Mao D., Khadaroo P.A., Su H. (2019). A meta-analysis of comparison of proximal gastrectomy with double-tract reconstruction and total gastrectomy for proximal early gastric cancer. BMC Surg..

[41] Chen X.-J., Chen G.-M., Wei Y.-C., Yu H., Wang X.-C., Zhao Z.-K., Luo T.-Q., Nie R.-C., Zhou Z.-W. (2021). Palliative Gastrectomy versus Gastrojejunostomy for advanced Gastric cancer with outlet obstruction: A propensity score matching analysis. BMC Cancer.

[42] Li J., Zeng J., Chen J., Shi J., Luo X., Fang G., Chai W., Zhang W., Liu T., Niu L. (2017). Evaluation of the safety of irreversible electroporation on the stomach wall using a pig model. Exp. Ther. Med..

[43] Lee J.M., Choi H.S., Kim E.S., Keum B., Seo Y.S., Jeen Y.T., Lee H.S., Chun H.J., Um S.H., Kim C.D. (2019). Characterization of irreversible electroporation on the stomach: A feasibility study in rats. Sci. Rep..

[44] Ren F., Li Q., Hu L., Yan X., Gao Z., Zhang J., Gao W., Zhang Z., Chang P., Chen X. (2020). Safety and efficacy of magnetic anchoring electrode-assisted irreversible electroporation for gastric tissue ablation. Surg. Endosc..

[45] Li Q., Gao X., Zhang Y., Han X., Li Z., Zhang Y., Wang Y., Liang L., Chu D., Wu Z. (2021). Magnetic anchoring and guidance-assisted endoscopic irreversible electroporation for gastric mucosal ablation: A preclinical study in canine model. Surg. Endosc..

[46] Jeon H.J., Choi H.S., Keum B., Bang E.J., Lee K.W., Kim S.H., Yim S.Y., Lee J.M., Kim E.S., Seo Y.S. (2021). Feasibility and effectiveness of endoscopic irreversible electroporation for the upper gastrointestinal tract: An experimental animal study. Sci. Rep..

[47] Ren W., Beebe S.J. (2011). An apoptosis targeted stimulus with nanosecond pulsed electric fields (nsPEFs) in E4 squamous cell carcinoma. Apoptosis.

[48] Chen R., Sain N.M., Harlow K.T., Chen Y.-J., Shires P.K., Heller R., Beebe S.J. (2014). A protective effect after clearance of orthotopic rat hepatocellular carcinoma by nanosecond pulsed electric fields. Eur. J. Cancer.

[49] Ren W., Sain N.M., Beebe S.J. (2012). Nanosecond pulsed electric fields (nsPEFs) activate intrinsic caspase-dependent and caspase-independent cell death in Jurkat cells. Biochem. Biophys. Res. Commun..

[50] Beebe S.J., Sain N.M., Ren W. (2013). Induction of Cell Death Mechanisms and Apoptosis by Nanosecond Pulsed Electric Fields (nsPEFs). Cells.

[51] Szewczyk A., Saczko J., Kulbacka J. (2020). Apoptosis as the main type of cell death induced by calcium electroporation in rhabdomyosarcoma cells. Bioelectrochemistry.

[52] Hall E.H., Schoenbach K.H., Beebe S.J. (2007). Nanosecond pulsed electric fields induce apoptosis in p53-wildtype and p53-null HCT116 colon carcinoma cells. Apoptosis.

[53] Ren Z., Chen X., Cui G., Yin S., Chen L., Jiang J., Hu Z., Xie H., Zheng S., Zhou L. (2013). Nanosecond pulsed electric field inhibits cancer growth followed by alteration in expressions of NF-κB and Wnt/β-catenin signaling molecules. PLoS ONE.

[54] Nuccitelli R., Chen X., Pakhomov A.G., Baldwin W.H., Sheikh S., Pomicter J.L., Ren W., Osgood C., Swanson R.J., Kolb J.F. (2009). A new pulsed electric field therapy for melanoma disrupts the tumor’s blood supply and causes complete remission without recurrence. Int. J. Cancer.

[55] Shi Y. (2002). Mechanisms of Caspase Activation and Inhibition during Apoptosis. Mol. Cell.

[56] Ramuz O., Isnardon D., Devilard E., Charafe-Jauffret E., Hassoun J., Birg F., Xerri L. (2003). Constitutive nuclear localization and initial cytoplasmic apoptotic activation of endogenous caspase-3 evidenced by confocal microscopy. Int. J. Exp. Pathol..

[57] Feng Y., Wu J., Feng X., Tao D., Hu J., Qin J., Li X., Xiao W., Gardner K., Judge S.I. (2007). Timing of apoptosis onset depends on cell cycle progression in peripheral blood lymphocytes and lymphocytic leukemia cells. Oncol. Rep..

[58] Saraste A., Pulkki K. (2000). Morphologic and biochemical hallmarks of apoptosis. Cardiovasc. Res..

[59] Faroja M., Ahmed M., Appelbaum L., Ben-David E., Moussa M., Sosna J., Nissenbaum I., Goldberg S.N. (2013). Irreversible electroporation ablation: Is all the damage nonthermal?. Radiology.

[60] van Gemert M.J.C., Wagstaff P.G.K., de Bruin D.M., van Leeuwen T.G., van der Wal A.C., Heger M., van der Geld C.W.M. (2015). Irreversible electroporation: Just another form of thermal therapy?. Prostate.

[61] Ben-David E., Appelbaum L., Sosna J., Nissenbaum I., Goldberg S.N. (2012). Characterization of irreversible electroporation ablation in in vivo porcine liver. AJR Am. J. Roentgenol..

[62] Tarnawski A., Szabo I.L., Husain S.S., Soreghan B. (2001). Regeneration of gastric mucosa during ulcer healing is triggered by growth factors and signal transduction pathways. J. Physiol. Paris.

[63] Ypsilantis P., Pitiakoudis M., Souftas V.D., Lambropoulou M., Tsalikidis C., Foutzitzi S., Tsigalou C., Prassopoulos P., Papadopoulos N., Simopoulos C. (2008). Liver regeneration following radiofrequency ablation. J. Surg. Res..

[64] Kakushima N., Fujishiro M., Kodashima S., Kobayashi K., Tateishi A., Iguchi M., Imagawa A., Motoi T., Yahagi N., Omata M. (2006). Histopathologic characteristics of gastric ulcers created by endoscopic submucosal dissection. Endoscopy.

[65] Herrera J., Henke C.A., Bitterman P.B. (2018). Extracellular matrix as a driver of progressive fibrosis. J. Clin. Investig..

[66] McQuitty C.E., Williams R., Chokshi S., Urbani L. (2020). Immunomodulatory role of the extracellular matrix within the liver disease microenvironment. Front. Immunol..

[67] Chen C., Bai X., Ding Y., Lee I.-S. (2019). Electrical stimulation as a novel tool for regulating cell behavior in tissue engineering. Biomater. Res..

[68] Johnson A., DiPietro L.A. (2013). Apoptosis and angiogenesis: An evolving mechanism for fibrosis. FASEB J..

[69] Schoenbach K.H., Katsuki S., Stark R.H., Buescher E.S., Beebe S.J. (2002). Bioelectrics-new applications for pulsed power technology. IEEE Trans. Plasma Sci..

[70] Sung C.K., Kim H.B., Jung J.H., Baik K.Y., Moon K.W., Kim H.-S., Yi J.-H., Chung J.H. (2017). Histological and mathematical analysis of the irreversibly electroporated liver tissue. Technol. Cancer Res. Treat..

[71] Ertel A., Verghese A., Byers S.W., Ochs M., Tozeren A. (2006). Pathway-specific differences between tumor cell lines and normal and tumor tissue cells. Mol. Cancer.

